# Hemicellulose-Derived Oligosaccharides: Emerging Prebiotics in Disease Alleviation

**DOI:** 10.3389/fnut.2021.670817

**Published:** 2021-07-27

**Authors:** Uttam Kumar Jana, Naveen Kango, Brett Pletschke

**Affiliations:** ^1^Department of Microbiology, Dr. Harisingh Gour Vishwavidyalaya (A Central University), Sagar, India; ^2^Department of Biochemistry and Microbiology, Rhodes University, Makhanda, South Africa

**Keywords:** gut microbiota, hemicelluloses, probiotic, oligosaccharide, nutrition

## Abstract

The gut microbiota in the human body is an important component that plays a pivotal role in the ability of the host to prevent diseases and recover from these diseases. If the human microbiome changes for any reason, it affects the overall functioning of the host. Healthy and vigorous gut microbiota require dietary fiber supplementation. Recently, oligosaccharides have been found to play a significant role in the modulation of microbiota. Several such oligosaccharides, i.e., xylooligosaccharides (XOS), mannooligosaccharides (MOS), and arabino-xylooligosaccharides (AXOS), are derived from hemicellulosic macromolecules such as xylan, mannan, and arabino-xylan, respectively. These oligosaccharides serve as substrates for the probiotic production of health-promoting substances (short-chain fatty acids, branched chain amino acids etc.), which confer a variety of health benefits, including the prevention of some dreaded diseases. Among hemicellulose-derived oligosaccharides (HDOs), XOS have been largely explored, whereas, studies on MOS and AXOS are currently underway. HDOs, upon ingestion, help reduce morbidities by lowering populations of harmful or pathogenic bacteria. The ATP-binding cassette (ABC) transporters are mainly utilized for the uptake of oligosaccharides in probiotics. Butyrate generated by the selective fermentation of oligosaccharides, along with other short-chain fatty acids, reduces gut inflammation. Overall, oligosaccharides derived from hemicelluloses show a similar potential as conventional prebiotics and can be supplemented as functional foods. This review summarizes the role of HDOs in the alleviation of autoimmune diseases (inflammatory bowel disease, Crohn's disease), diabetes, urinary tract infection, cardiovascular diseases, and antimicrobial resistance (AMR) through the modulation of the gut microbiota. The mechanism of oligosaccharide utilization and disease mitigation is also explained.

## Introduction

Humans are very interested in controlling and minimizing the risk of their personal health, and modern consumers have a strong preference for natural food over processed food (1). An adequate consumption of dietary short-chain carbohydrates, such as dietary fibers and oligosaccharides, decreases the risk of development of diseases such as colorectal cancer (CRC), cardiovascular diseases, obesity, diabetes, etc. (2). The human gut comprises ~10^13^-10^15^ microbial cells that amount to a complex and diverse microbial community. Host gut microbiota vary among individuals and have a close relation with different factors such as the sex, age, health, diet, genetic makeup, and immune system of the host. However, Firmicutes, Bacteroidetes, and Actinobacteria comprise the most common microbiota residing in the human gut (3). Together, these communities contain much more genomic information (100-fold) than the host itself, which leads to functional expansion of the abilities of the host. Functional oligosaccharides directly influence the gut microbiota and help to produce different key health-promoting metabolites, which are directly associated with the physiology of the host (4, 5). For this reason, the physicochemical and physiological properties of non-digestible carbohydrate fibers have drawn the attention of food scientists who explore them as functional food ingredients. Different types of oligosaccharides, like galactooligosaccharides (GOS), fructooligosaccharides (FOS), and inulooligosaccharides (IOS), are already recognized as nutraceuticals and are frequently used in synbiotic pharmaceutical preparations. Hemicellulose-derived oligosaccharides (HDOs) such as XOS and mannooligosaccharides (MOS) are rapidly emerging prebiotics, which fall in this category and have similar bioactive properties as conventionally used oligos (FOS) (6). XOS and MOS are easily produced from different low-value substrates (locust bean gum, guar gum, and konjac gum), as well as different agro-wastes (corn cob, copra meal, palm kernel cake, and corn cob), by enzymatic hydrolysis using hemicellulases such as endo-β-(1 → 4)-xylanase and endo-β-(1 → 4)-mannanase (7, 8). These oligosaccharides contribute to the important physiological functions of dietary fibers: (1) Their consumption does not increase the blood glucose level or spike the secretion of insulin because of the formation of a gel in the gut through which it dissolves, (2) their nature is non-cariogenic and low calorific (0–3 kcal/g of sugar), (3) they stimulate the growth of specific microorganisms that enrich the gut environment by decreasing pH, and (4) they ameliorate the absorption of the minerals (mainly calcium) through the intestinal cells. Thus, HDOs work as a silent health promoter, thus lowering the risk of different complex health issues (9, 10).

From a commercial point of view, the oligosaccharide market is increasing rapidly and is expected to reach a total turnover of 7.37 billion USD by 2023. In the case of XOS, the market is growing at a rate of ~4.1% per annum and is expected to reach a projected value of 130 million USD by 2025 from 94 million USD in 2018 (11). This high growth is mainly because of the advanced scientific research and continuous development in the field of oligosaccharides and their product development. These oligosaccharides are also being utilized in pharmaceuticals, feeds, cosmetics, and as immunostimulating agents and bulking agents (12). Some of the commercially produced oligosaccharides and their uses are indicated in Table 1. The nutritional significance of oligosaccharides (including HDOs) has been indicated in various diseases, like heart infections, autoimmune diseases, osteoporosis, and many chronic diseases. Conventional oligosaccharides such as FOS are well-documented, while HDOs are emerging and scientists are trying to establish their role in the amelioration of diseases. This review summarizes the biochemical properties of HDOs and their impact in reducing the development of diseases through the modulation of gut microbiota.

**Table 1:** Some of the commercially produced prebiotic oligosaccharides and their uses.

**Oligosaccharides/commercial name**	**Specifications/composition**	**Commercial producer**	**Application(s)**
Frutalose®	Fructooligosaccharide	Sensus, United States	Better sweetener and helps to restore and maintain a balanced microflora
Orafti®P95	Fructooligosaccharide	Beneo GmbH, Germany	Applied as a natural sugar replacer
Frutafit®	Inulin, fructose, glucose	Sensus, United States	Probiotic effect and microbiome modulation
Oligomate 55	Galactooligosaccharides, lactose, glucose, galactose	Yakult Honsha Tokyo, Japan	Functional sweetener and probiotic effect
XOS Prebiotic (XOS)	Xylooligosaccharide	Van Wankum Ingredients, the Netherlands	Serve as a food supplement
PreneXOS™	Xylooligosaccharide	Prenexus Health, United States	Minimize side effects of bloating and improve the overall gut health
XOS95P	Xylooligosaccharide	Shandong Longlive Bio-Tech Co., Ltd, China	Use as a functional sugar
Bio-Mos®	Mannooligosaccharides	Alltech, United States	Upgrade animal feed
ActiveMOS®	Mannooligosaccharides	Orffa, the Netherlands	Use as an animal feed additive
AgriMOS	Mannooligosaccharides	Lallemand Inc., Canada	Use as a feed ingredient

## Gut Microbiota and HDOs

Gut microbiota are a collective environment of microorganisms, like bacteria, fungi, viruses, and protozoans present in the gastrointestinal tract (GIT) (13). These microorganisms act as regulators of the metabolism of the host. The role of gut microbiota in disease control has drawn a significant attention over the past few decades, which were kept hidden for a long time in the absence of metagenomic techniques and suitable cultivation media. Currently, an analysis of the collective genome of the gut microbiota through the next-generation sequencing has helped us in unraveling complex gut ecosystem (14). Gut microbiota of humans start developing in the fetus itself and are strongly influenced by the microbiome of the mother. Alterations in the microbiome completely depend upon several factors, such as the process of parturition, surrounding environment, infant feeding method, lifestyle, stress, and diet. The key taxa involved in the gut microbial diversity of individuals include *Lactobacillus, Ruminococcus, Bifidobacterium, Clostridium, Eubacterium, Akkermansia, Butyrivibrio, Roseburia, Prevotella, Faecalibacterium, Bacillus, Oxalobacter, Lachnospiraceae*, and *Blautia* (15). Breast milk contains higher amounts of *Bifidobacterium* and *Lactobacillus*; therefore, infants on high-breast-milk diet tend to have a greater preponderance of the two probiotic species (16). In addition, the Firmicutes/Bacteroidetes ratio may be an important biomarker in the case of humans that exhibit morbidity (17, 18). Thus, it may be concluded that gut microbiota play a pivotal role in human physiology and have an impact on the alleviation of diseases for better health. The gut microbiota can be modulated favorably using prebiotic oligosaccharides. Apart from the frequently used FOS, and more recently, HDOs have been found to confer a selective advantage to gut microbiota (Table 2). The intake of HDOs was found to diversify the human gastrointestinal microbiota and increase the defense against different chronic non-communicable diseases (28). XOS were found to reduce gut disturbance, as well as gut inflammation, by lowering the Firmicutes/Bacteroidetes ratio and Enterobacteriaceae in obese rats (20). XOS have also been indicated in resisting weight gain by increasing the population of *Bifidobacteria* and *Lachnospiraceae* in cecum microbiota (29), while MOS have been found to reduce the gut inflammation by decreasing the *Clostridium* content in gut microbiota of piglets (30).

**Table 2:** Role of HDOs in health promotion and gut microbiota modulation.

**HDOs**	**Health benefits or Disease alleviation**	**Gut microbiota modulation**	**References**
XOS	Reduce the visceral fat cells	Lowering the population ratio of Firmicutes and Bacteroidetes	(19)
	Decrease the metabolic endotoxemia	Reduce the population ratio of Firmicutes and Bacteroidetes	(20)
	Reduce development of diabetes	Increase the abundance of *Blautia hydrogenotrophica*	(21)
	Reduce the antibiotic-associated diarrhea	Increase the population of *Bifidobacteria*	(22)
	Make unfavorable conditions for colorectal cancer-risking pathogen	Induce the major genera, mainly *Bifidobacterium* spp., *Lactobacillus* spp.,	(23)
MOS	Reduce inflammation of dextran sulfate sodium-induced colitis	Induce the growth of coliform bacteria	(24)
	Reverse high-fat-diet-induced disorder	Lowering the population ratio of Firmicutes and Bacteroidetes	(25)
	Ameliorate insulin resistance and glucose tolerance	Increase *Akkermansia muciniphila* and *Bifidobacterium pseudolongum* population and decrease *Rikenellaceae* and *Clostridiales* density	(26)
AXOS	Decreased insulin resistance	Induced the population of *Bifidobacterium* spp., *Lactobacillus* spp., *Bacteroides-Prevotella* spp.	(27)

## Types of HDOs

The two major plant hemicelluloses are xylan and mannan, and accordingly, XOS and MOS make up most of the potential HDOs. Due to the diverse and heteropolymeric nature of xylans and mannans, the nature of derived oligosaccharides varies. In general, a repertoire of hydrolyzing enzymes is employed to achieve the controlled degradation of the hemicelluloses.

### Xylooligosaccharides

**Figure 1:**
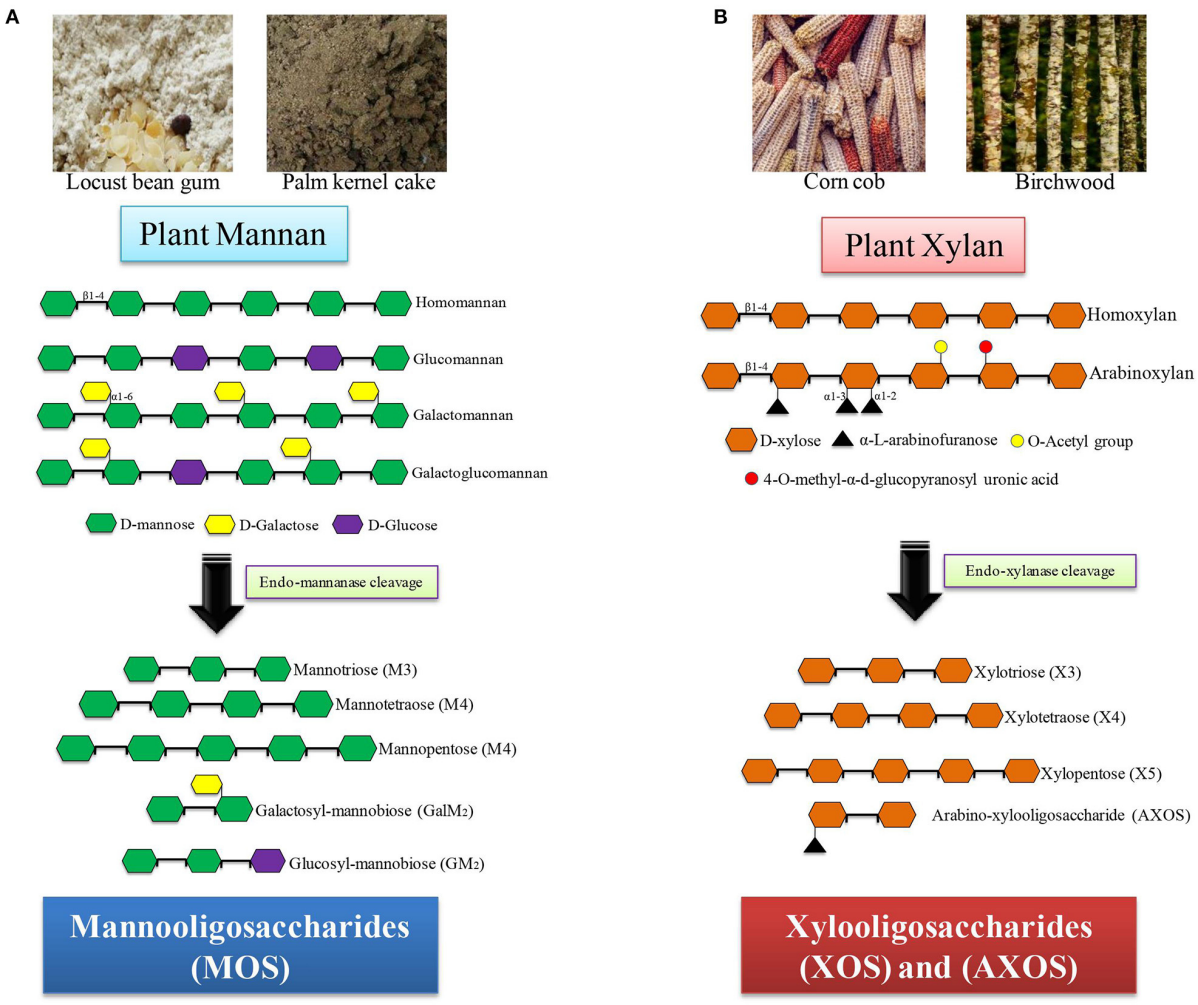
Sources and structure of hemicelluloses-derived oligosaccharides (HDOs). **(A)** Mannan containing substrates (locust bean gum, palm kernel cake) produce different mannooligomers after mannanase treatment. **(B)** Xylan containing substrates (corn cob, birchwood) produce xylooligomers after xylanase treatment.

Xylooligosaccharides are short oligomers composed of xylose moieties and are commonly derived from corn cob, bamboo shoots, wheat straw, sugarcane bagasse, and hardwood xylan. Among these, corn cob contains the highest amount of xylan (35–38%), and hence, it can be exploited as a cheap and renewable source of XOS (31). Based on the sugar units and linkages present, the exact composition and the quantity of the produced XOS vary from one plant source to another. XOS generated after the hydrolysis of xylan are short-chain XOS and consist of xylobiose, xylotriose, and xylotetraose. XOS can be produced by the action of xylan-degrading enzymes such as endo-β-1,4-xylanase (EC 3.2.1.8) and xylan 1, 4-β-xylosidase (EC 3.2.1.37), with the help of other side chain-degrading enzymes, like α-glucuronidase (EC 3.2.1.139), acetyl xylan esterase (EC 3.1.1.72), α-L-feruloyl esterase (EC 3.1.1.73), and arabinofuranosidase (EC 3.2.1.55) (32). The sources and structural details of xylan-derived MOS are presented in Figure 1.

### Mannooligosaccharides

Mannooligosaccharides are short chains of repeating units of mannose linked by glycosidic bonds. Based on the source, the MOS are divided into two major groups, namely, α- and β-MOS. α-MOS are commonly obtained from the physicochemical hydrolysis of the cell wall of the yeast (*Saccharomyces cerevisiae*). α-MOS are commonly used in the animal agriculture sector as a feed additive. β-MOS are plant-derived MOS obtained after enzymatic, alkaline, or acidic hydrolysis of plant β-mannan and are commonly found in locust bean gum, konjac gum, and guar gum. β-MOS can be generated by applying a combination of β-1, 4-mannanase (EC 3.2.1.78), β-mannosidase (EC3.2.1.25), α-galactosidase (EC 3.2.1.22), and β-glucosidase (EC 3.2.1.21) for the hydrolysis of plant mannans such as LBG, GG, and KG (33, 34). The sources and structural details of mannan-derived MOS are given in Figure 1.

## Mechanism of Transport of Oligosaccharides

**Figure 2:**
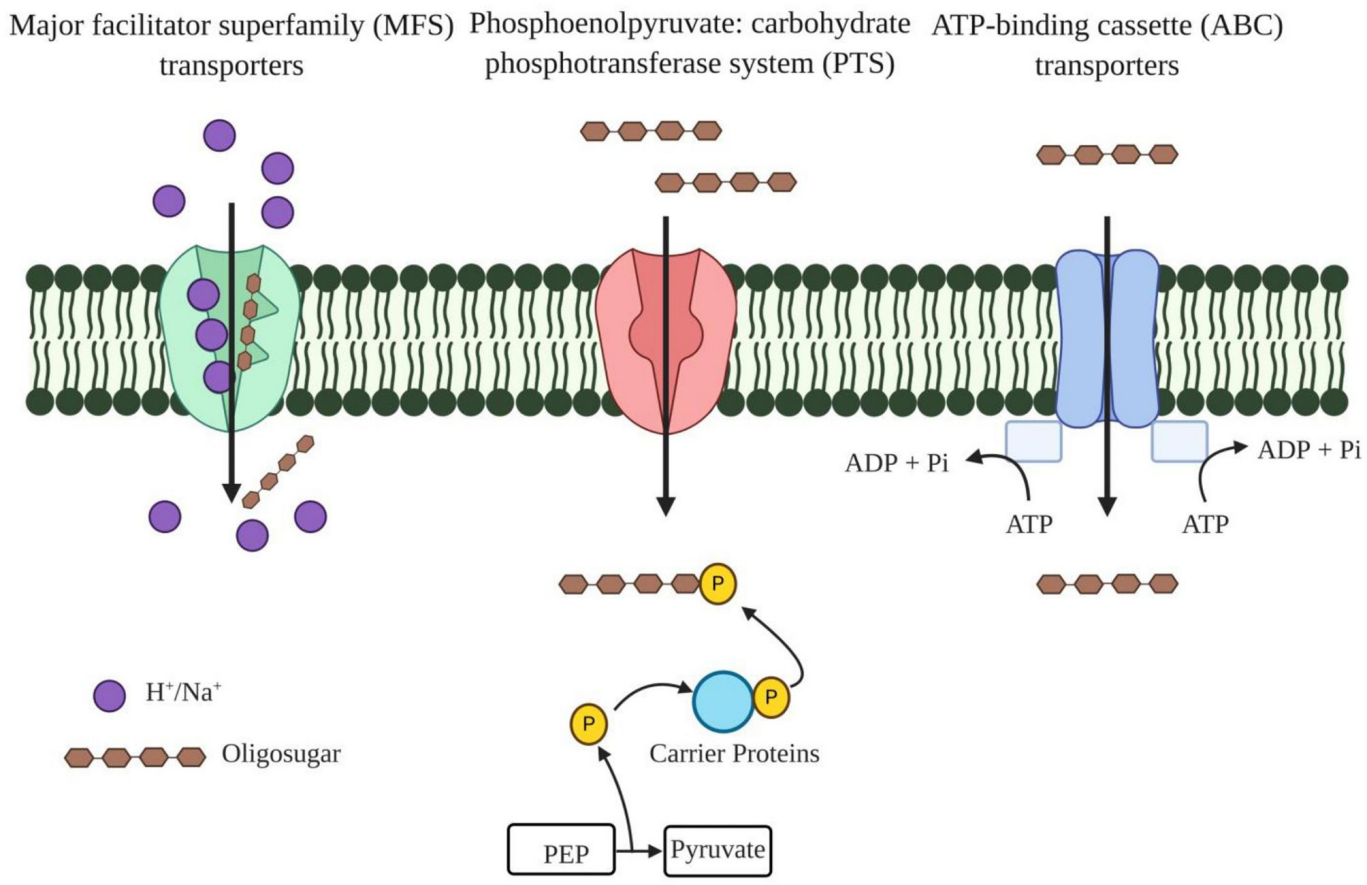
Different oligosaccharide transporters present in the plasma membrane of bacteria. Major facilitator superfamily (MFS) transporters function as a symporter that transport oligosaccharide alongside with Na^+^/H^+^ as a proton motive factor. Phosphoenolpyruvate (PEP): carbohydrate phosphotransferase system (PTS) transports the oligosaccharide and transported oligosaccharides are further phosphorylated to diffuse back from the cell. ATP binding cassette transporters (ABC) have nucleotide binding domains (NBDs) as these power the transport of substrates across cell membranes by ATP-binding and hydrolysis.

Probiotic bacteria supplemented with prebiotic oligosaccharides are reported to impart several health benefits, including improved immunity and relief in gastrointestinal disorders, but the precise route of specific oligosaccharide uptake is yet to be determined. According to reports available thus far, three main transport systems, such as ATP-binding cassette (ABC) transporters, major facilitator superfamily (MFS) transporters, and the phosphoenolpyruvate (PEP): carbohydrate phosphotransferase system (PTS) (Figure 2), are used to transport these oligosaccharides (35). ABC transporters import oligosaccharides by utilizing energy from ATP hydrolysis and are regulated by the two-component systems (TCSs), alongside the involvement of a different gene cluster for polymer hydrolysis (Table 3) (43). The drivers of ABC transporters are the nucleotide-binding domains (NBDs) that conduct the transport of substrates across cell membranes by ATP binding and hydrolysis. The xyloside ABC transporter functions as a membrane permease that promotes the transport of XOS across the cell membrane (36). The ATPase present in the ABC transporter has several domains that interact with more than one ABC transporter, and it simultaneously functions with transporters that form a complex network of ABC transporters (44). *Streptomyces thermoviolaceus* has been shown to have two integral ABC transporters in the cell membrane containing a conserved EAA (glutamic acid-alanine-alanine) domain. These two proteins are regulated by a transcriptional regulator protein that directs the uptake of XOS along with the degradation the xylan polymer (45). A MFS transporter acts as a symporter like XOS in bacteria and performs the transport through xyloside/Na^+^ (H^+^) symporters. Xyloside/Na^+^ (H^+^) symporters are the multipass transmembrane proteins in the cell membrane that transport XOS alongside with sodium as a proton motive force (36). The PTS operates through a facilitated diffusion where imported sugar is phosphorylated and the modification does not allow these sugars to diffuse back from the cell. During evolution, the PTS has emerged in the late bacterial evolution stage and, therefore, could not be found in many early bacterial lineages. It is found mainly in lactic acid bacteria and enterobacteria. In particular, homo-fermentative lactobacilli have significant PTS transporters compared to hetero-fermentative lactobacilli (46).

**Table 3:** Gene clusters and enzymes involved in polysaccharide utilization by microorganisms present in the gut.

**Organism**	Gene cluster/locus^*^	**Type of transporter**	**Enzymes involved**	**Types of oligosaccharide**	**References**
*Corynebacterium alkanolyticum*	xylEFGD	ABC transporter	β-Xylosidase α-L-Arabinofuranosidase	XOS AXOS	(36)
*Roseburia intestinalis*	MUL	ABC transporter	Acetyl esterases α-Galactosidase β-Glucosidases Exo-oligomannosidase	β-MOS	(37)
*Bifidobacterium animalis* subsp. *lactis* ATCC27673	MUL	ABC transporter	β-Mannosidase β-Glucosidase	β-MOS	(38)
*Faecalibacterium prausnitzii*	MUL	ABC transporter	α-Galactosidase Carbohydrate esterases Epimerase β-1,4-Mannooligosaccharide phosphorylase Mannosylglucose phosphorylase Phosphomutase Isomerase	β-MOS	(39)
*Bacteroides ovatus* ATCC 8483	PUL	–	β-Mannanase	β-MOS	(40)
*Prevotella copri* DSM18205	PUL10 PUL15	–	β-1,4-Xylanase α-L-Arabinofuranosidase β-1,4-Xylosidase α-Glucuronidase β-Galactosidase	XOS	(41)
*Bacteroides xylanisolvens* XB1A^T^	PUL43 PUL70	ABC transporter	Endo-xylanase	XOS	(42)

* *PUL, polymer-utilizing locus; MUL, mannan-utilizing locus*.

The β-MOS ABC transporter in *Bifidobacterium animalis* has two oligosaccharide-specific extracellular lipid-anchored solute-binding protein (SBP) genes. This transporter recognizes the mannosyl unit of MOS at position 2 through the asparagine and glycine amino acids, respectively (38). La Rosa et al. (37) reported that the Firmicute, *Roseburia intestinalis*, has an ABC transporter containing three subunits that transported the smaller MOS from all types of mannans (glucomannan, galactomannan, acetyl-galactoglucomannan, and undecorated mannan), which were further completely hydrolyzed by the intracellular enzyme cocktail. Unlike the sugar/cation symporters, mannoside/Na^+^ (H^+^) symporters were also noticed in *Bacteroides fragilis* that transferred MOS into the bacterial cell (47).

## Mode of Action of Oligosaccharides Through SCFAs

**Figure 3:**
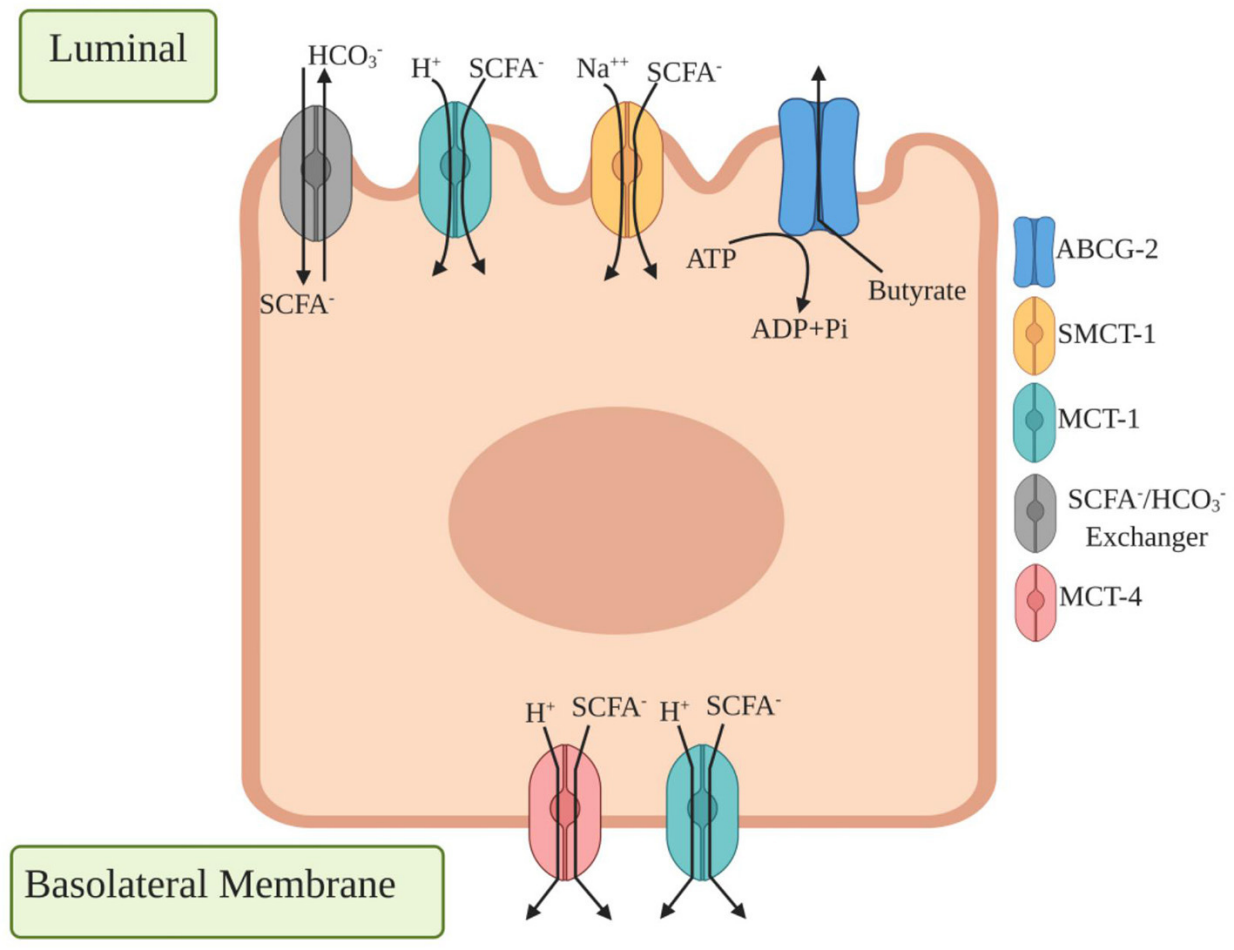
Different transporter proteins expressed in colonic epithelium cells for transport of short chain fatty acids (SCFA). Luminal side expressed transporters are ${\text{HCO}}_{3}^{-}$/SCFA^−^ exchangers which secrete ${\text{HCO}}_{3}^{-}$ that result in SCFA^−^ transport across the apical membrane, monocarboxylate transporter 1 (MCT1) binds a proton first followed by monocarboxylates such as lactate, pyruvate, and transport to the cell where basolateral side expressed monocarboxylate transporter 4 (MCT4) functions similarly by exporting SCFA^−^ from the basolateral membrane. ATP binding cassette transporters G family Member 2 (ABCG2) is expressed in apical colonic cells where they bind with butyrate as a substrate for efflux in intestinal cells.

Different studies have concluded that oligosaccharides prevent gut damage caused by pathogenic bacteria or a diet containing harmful chemicals such as lectin (48). The oligosaccharides enhance the population of probiotic bacteria and increase the production of short chain fatty acids (SCFA's), including acetate, propionate, and butyrate (7, 49). These acids have an important role in increasing the transepithelial fluid transport and epithelial defense barrier and in decreasing mucosal inflammation and oxidative stress. They play a significant role in hypercholesterolemia, insulin resistance, hemoglobinopathies, and genetic metabolic diseases. SCFAs are absorbed and utilized by the enteric cells as the main source of energy (50). The transportation of the SCFAs in the colonic epithelium and mucosal immune cells is directed through different transporters like monocarboxylate transporters, G-protein-coupled receptors (GPCRs), intracellular receptors, and several other enzymes. The main transporters involved in SCFA transportation are monocarboxylate transporters 1 and 4 (MCT-1 and MCT4), which are basically proton-coupled transporters, e.g., sodium-coupled monocarboxylate transporter 1 (SMCT-1) (Figure 3). The MCT-1 is expressed in both the apical and basolateral sides of the colonic cells, while MCT-4 is found at the basolateral side (Figure 3). SCFAs are used as ligands by many GPCRs, mainly GPR41, GPR43, and GPR109A for initially different signaling cascades. Other two less explored transportation mechanisms are through the ABC superfamily G member 2 (ABCG2) and SCFA^−^/${\text{HCO}}_{3}^{-}$ exchanger. ABCG2 is expressed in the apical colonic cells where it binds with butyrate as a substrate for efflux in the intestinal cells. The SCFA^−^/${\text{HCO}}_{3}^{-}$ exchanger is present in the small intestine and colon, and the secretion of ${\text{HCO}}_{3}^{-}$ leads to an increased uptake of SCFA^−^ into the vesicles (51, 52). Butyrate has an inhibitory effect on histone deacetylase (HDAC), which leads to the modulation of different oncogenic signaling pathways such as the JAK2/STAT3 and VEGF pathways, and it can also influence the extrinsic apoptotic and mitochondrial apoptotic pathways. Butyrate is shown to relieve gut inflammation by regulating the T_reg_ cell differentiation and NF-κB and STAT3 pathways (53, 54).

## Role of HDOs in Disease Alleviation

### Cardiovascular Diseases

Cardiovascular diseases are a major health concern in low- and middle-income countries causing an estimated 31% of all the global deaths (https://www.who.int/news-room/fact-sheets/detail/cardiovascular-diseases-(cvds). The abnormality of lipid content in serum is a biomarker of cardiovascular risk, which is influenced by daily diet components. Diets with plant-based food such as “Mediterranean diet” and “Prudent diet” have been shown to be protective compared to the “Western diet” (55). The high-fat diet and low fiber intake result in the Western diet higher growth of Firmicutes and lowering of Bacteroidetes in the gut, which is an important marker of obesity (56). Different studies using different model organisms showed that plant-derived, non-digestible oligosaccharides, such as XOS and MOS, have a high impact on serum lipoprotein and cholesterol levels, which compensate for the higher risk of cardiovascular disease (57). XOS supplementation reduced lipogenesis by decreasing the activity of different lipogenic enzymes such as fatty acid synthetase, malic enzyme, and others (58). On the other hand, it also upregulated the lipoprotein lipase, which was responsible for breaking down fat in the form of triglycerides (lipolysis) (59). Moreover, the XOS diet mainly reduced the visceral fat cells by downregulating the *Mcp* 1 gene, rather than the blood and liver fat cells, by lowering the population ratio of obesity-related microbiota Firmicutes and Bacteroidetes (19). XOS, especially xylobiose, was able to downregulate the lipogenic and adipogenic genes in mesenteric fat and liver in case of a high-fat-diet-supplemented mice (60). A high-fat diet induced macrophage infiltration in adipose tissue, insulin resistance, metabolic endotoxemia, inflammatory stress, and other biochemical issues in the body system. AXOS treatment in diet-induced obese mice altered the gut microbiota, resulting in higher levels of appetite suppressing satietogenic peptides, which reduced macrophage infiltration in the adipose tissues (27).

### Obesity

Obesity is a metabolic disorder that occurs due to excessive fat accumulation in the body after being fed a high-fat diet. This lifestyle disease is a result of energy-balance dysregulation, an unhealthy sedentary routine, and also individual genetic traits (25). According to the WHO, 1.9 billion adults were found to be overweight, and among these, over 650 million adults were obese. In 2016, data showed that 13% of the adult populations (11% of men and 15% of women) of the world were obese. The increase in obesity nearly tripled between 1975 and 2016, which is not only a problem in high-income countries but also a rising problem in low- and middle-income countries (https://www.who.int/news-room/fact-sheets/detail/obesity-and-overweight). A healthy diet could modulate the gut microbiota, which supported a loss in body weight. It was reported that an abundance of a particular bacterial group like Firmicutes/Bacteroidetes had a positive effect in overcoming obesity in mice. Different studies have indicated that the gut microbiota can be modulated by different oligosaccharides, which have a preventive effect on obesity. MOS intake could reverse the high-fat-diet-induced disorder by restructuring the overall composition of the gut microbiota, including lowering the Firmicutes/Bacteroidetes ratio (25). Coffee-based MOS, either as a mixture or in the pure form, had a different effect on the fat deposition in the tissue. The MOS mixture was reported to decrease the gain in body weight, body fat, and visceral adipose tissue, whereas, purified MOS had no such effect on these parameters (61). Prebiotic oligosaccharides were shown to have gender-specific activity in the reduction of fat. In a study, men consuming an MOS containing beverage experienced a greater weight loss than their female counterparts, which signified the importance of MOS for strategic weight management and improvement in adipose tissue distribution (62). MOS can also control excess appetite-causing genes and modulate the expression of appetite-related hormones like leptin, proopiomelanocortin (POMC), cocaine- and amphetamine-regulated transcript (CART), and neuropeptide Y (NPY) (63). XOS consumption decreased metabolic endotoxemia and reduced the Firmicutes/Bacteroidetes ratio in the obese rat (20). Monocyte chemoattractant protein 1 (MCP-1) is one of the major proteins found in white adipose tissue that is overexpressed in the obese compared to those persons of proper weight. MCP-1 helps to differentiate the adipocyte, which decreases insulin-stimulated glucose uptake (64). XOS significantly reduced the plasma levels of MCP-1 by lowering the mRNA expression in high-fat diet-induced obesity (65).

### Type 2 Diabetes

Type 2 diabetes is a chronic metabolic condition that arises when pancreatic β-cells lose their function (66, 67). According to the IDF (International Diabetes Federation) Diabetes Atlas (9th edition, 2019), ~463 million adults aged between 20 and 79 years have diabetes, and it will increase to 700 million by 2045. Among these, 374 million people will have the risk of developing Type 2 diabetes (https://www.idf.org/aboutdiabetes/what-is-diabetes/facts-figures.html). There is a significant role of the gut microbiota in managing Type 2 diabetes. Previous reports suggested that members of the genera *Roseburia, Faecalibacterium, Bacteroides*, and *Akkermansia* reduced T2D, whereas, *Fusobacterium, Ruminococcus*, and *Blautia* had a positive association with T2D (68). XOS derived from rice husk reduced insulin resistance and signaling and enhanced glucose uptake by altering the gut microbiota and mitigating endotoxemia. Glucose transporter 4 (GLUT-4) is known as the major glucose-transporting protein that promotes glucose uptake into the skeletal muscles and also controls glucose homeostasis in the body. XOS administration decreased the expression of GLUT-4 and transferred it from the cytosolic compartment to the plasma membrane by Akt (protein kinase B) activation through phosphorylation (69). Prediabetic adults had a higher percentage of *Howardella, Enterorhabdus*, and *Slackia* populations in their stools. XOS treatment increased the abundance of positively associated microflora species such as *Blautia hydrogenotrophica* (21). Similarly, MOS obtained from konjac glucomannan modulated the gut microbiota by increasing the population of *Akkermansia muciniphila* and *Bifidobacterium pseudolongum*, while decreasing *Rikenellaceae* and *Clostridiales* density (26). At the molecular level, MOS improved glucose and insulin tolerance by modulating the insulin signaling pathway through the activation of GLUT-2 and its translocation into the membrane. MOS also upregulated the expression of leptin-associated protein and downregulated the negative regulators of the insulin signaling pathway proteins, protein tyrosine phosphatase 1B, and suppressor of cytokine signaling 3 (70).

### Autoimmune Diseases [Inflammatory Bowel Disease]

Inflammatory bowel disease covers two major diseases [Crohn's disease (CD) and ulcerative colitis (UC)], which severely affect the GI tract, resulting in chronic and relapsing conditions. In the diseased state, patients with CD face intense inflammation in the GI tract, whereas, UC results in tissue damage of the deep areas of the colon and rectum (71). According to the Center for Disease Control and Prevention, 3 million (1.3%) US adults were diagnosed with IBD in 2015 (https://www.cdc.gov/ibd/data-statistics.htm). The main etiology in dysbiosis of gut microbiota is the reduction in community of commensal microorganisms due to an abnormal T-cell-mediated immune response (72). Several studies have demonstrated that supplements such as non-digestible carbohydrate fibers in a diet can enhance the growth of selective commensal microorganisms through anaerobic fermentation and attenuate the disease complications by alleviating gut inflammation. The fiber supplementation also improved the disrupted cell morphology, which developed due to the diseased state of the colon. Hemicellulose-derived XOS not only reduced the inflammation, but also helped to maintain the colon crypt cell integrity in attenuated chronic colitis in rats (73). These oligomers are fermented with varied efficiency depending upon the gut microbiota diversity. β-Diversity of the gut microbiota in patients with UC was significantly promoted by XOS treatment, whereas, α-diversity could not utilize and ferment XOS (74). In CD, different related genes and proteins like nucleotide-binding oligomerization domain-containing protein 2 (NOD2), immunity-related GTPase family M (IRGM), autophagy-related 16 Like 1 (ATG16L1), the toll-like receptor 4 (TLR4), and proinflammatory cytokines (IL-10, IL-1a, IL-1b) were found to be present, while the deterioration of α- and β-diversity of gut microbiota was also recorded (72). Treatment with synbiotic XOS and *Bifidobacterium infantis* downregulated the proinflammatory cytokines (TNF-α and IL-1β) and upregulated the anti-inflammatory cytokines (IL-10) in the colon cells of a dextran sodium sulfate-induced mouse. XOS supplementation significantly enhanced the expression of different junction proteins, including claudin-1 tight junction (TJ), zonula occludens-1 (ZO-1), and occluding junction in the colon tissue (75). Apart from XOS, MOS have also been found to alleviate IBD symptoms. MOS administration to dextran sulfate sodium-induced colitis mice enhanced the growth of coliform bacteria and lowered the expression of different proinflammatory cytokines (IL-5, IL-1a, IL-1b, G-CSF, and MCP-1). Also, MOS normalized the expression of muc2 (intestinal mucin) in the goblet cells of the colon and small intestine (24).

### Colorectal Cancer

Cancer is an abnormal growth of normal cells in any site of the body. It is the cause of most deaths globally with an estimate of 9.6 million in 2018 (https://www.who.int/news-room/fact-sheets/detail/cancer). CRC is one of the major causes of death and arises due to an increased intake of animal-based food diet rather than a plant-based diet (76). A plant-based diet contains high amounts of non-digestible short-chain carbohydrates that retard the growth of CRC by either fermenting it with the help of gut microbiota or directly binding to the cell surface receptor (77). XOS reduced 1, 2-dimethylhydrazine (DMH)-induced artificial colon cancer by activating glutathione-S-transferase and catalase present in the liver and colonic mucosa (78). The fermentation of oligosaccharides resulted in the production of metabolites such as SCFAs, which lowered the pH of the gut and produced the unfavorable condition of CRC-risking pathogens by enhancing the growth of lactobacilli and bifidobacteria. SCFAs also lowered the carcinogenic products and suppressed the bacterial conversion of pro-carcinogens to carcinogens (23). Among the SCFAs, butyrate was more lethal to CRC cells and inhibited their growth by inducing histone hyperacetylation and blocking the histone deacetylase (79). The synbiotic approach was useful in this case—a XOS and *Weissella cibaria* combination acted differently, where XOS significantly increased the acidification rate, while *W. cibaria* reduced cancer cell proliferation by inhibiting the different proteins, namely, TLR4, MyD88, MD2, and NF-κb (80). It was also reported that in some instances, XOS had a more pronounced inhibitory effect on the precancerous colon lesions than conventional oligosaccharides such as FOS and decreased the amount of aberrant crypt foci in the colon (81).

### Diarrhea

Diarrhea is a diseased state when colon cells are unable to absorb fluid sufficiently. Secretory diarrhea mainly results in lower absorption of essential ions due to infection and the release of toxins by pathogenic microorganisms and the depletion of beneficial microflora (82). Approximately 88% of diarrheal patients die because of insufficient hygiene, unsafe water, and inadequate medication. Among these patients, rotavirus infections in children below 5 years cause about 40% of hospitalizations for diarrhea (https://www.cdc.gov/healthywater/pdf/global/programs/globaldiarrhea508c.pdf). Nutraceuticals such as oligosaccharides may play an important role in alleviating diarrhea by selectively enhancing the population of favorable microorganisms (9). XOS lead to a significant increase in the *Bifidobacterium* population and also enhance the total counts of anaerobic bacteria and *Bacteroides fragilis* (83). Similarly, a synbiotic containing *Lactobacillus paracasei* with XOS and arabinogalactan significantly reduced diarrhea compared to the placebo group in children (84). A healthy human gut usually has a low bacterial translocation (BT) but extensive pathogen invasion increases the BT, which leads to a disruption of the gut barrier function (85). Consumption of AXOS not only increased the population of bifidobacteria in the gut but also decreased the disorders linked with antibiotic-associated diarrhea. In addition, AXOS intake enhanced the butyrate production in the colon, which further maintained the gut biological barrier function (22). In addition, a high intake of fermentable but poorly digestible carbohydrates has also been shown to confer negative effects to humans and animals. A high intake of XOS induced diarrhea due to subchronic oral toxicity and carbotoxicity (86). In general, high XOS consumption in human subjects was responsible for transient gastrointestinal discomfort (87).

### Urinary Tract Infections

Urinary tract infections are the most common infections occurring in the urinary system, including the urethra, bladder, ureters, or kidneys. UTIs are diagnosed in over 150 million people worldwide each year. UTIs are significantly more common in infants, older men, and women of all ages (88). Among these, it has been reported that women are most affected with a 12.6% annual incidence of this disease, compared to men showing only 3% annual incidence (89). The main causative agent for the disease is uropathogenic *Escherichia coli* (UPEC), and 70–80% cases of UTIs also showed the presence of *Staphylococcus, Klebsiella*, and *Enterococcus* species (90, 91). These pathogens make up the first line of infection through binding of the oligosaccharides present on the epithelial layer with the help of their own carbohydrate-binding proteins, and therefore, the presence of external oligosaccharides will be the first-line defense (92). Among all the HDOs, arabino-xyloglucan oligosaccharides have been found to be effective in the prevention of UTIs. Cranberry (*Vaccinium macrocarpon*) juice containing arabino-xyloglucan oligosaccharides showed an anti-adhesion effect against P-fimbriated *E. coli* in swine (93). The oligosaccharides have an adverse effect on different surface proteins which are involved in substrate translocation, including sugar across the bacterial cell membrane (94). In another study, high mannose-containing glycoprotein oligosaccharides attached to the pathogenic fimbriated *E. coli* were found to be the reason for mannose-inhibited hemagglutination (95).

### Antimicrobial Resistance

Antimicrobial resistance is acquired when pathogenic microorganisms develop the ability to overcome drug treatment. AMR is also frequently termed as antibiotic-resistant infection. According to the CDC, every year about 2.8 million people are diagnosed with infection of antibiotic-resistant bacteria. The first case of AMR was identified in 1942 with penicillin-resistant *Staphylococcus aureus* as the causal organism. There are three ways a microorganism can develop AMR: (1) selective pressure, where selective antimicrobial gene carrying survivors dominate the population after sudden treatment with antimicrobial agents; (2) mutations in different genes help a microorganism to survive against antimicrobial agents; and (3) horizontal gene transfer, where a drug resistance gene gets transferred to the non-drug-resistant microorganism. *Salmonella enteritidis* is one of the major causal agents of salmonellosis in birds and mammals. Multidrug-resistant phenotypes of *S. enteritidis* enter the human body *via* animal food consumption through the food chain. For this reason, poultry animals infected with *S. enteritidis* are a major source of infection in humans. Feed supplementation with MOS and XOS reduces *S. enteritidis* in poultry animals by increasing specific health-promoting microorganisms (96). MOS increased the population of *Bifidobacterium* spp., while decreasing the populations of Enterobacteriaceae members and *Enterococcus* spp. The increased population of Bifidobacteria produces SCFAs that reduce the pH. XOS promoted the growth of diverse groups of commensals like *Lactiplantibacillus* and *Levilactobacillus*, to produce high amounts of organic acids, which had an antimicrobial effect on both pathogenic bacteria and fungi (97). It has been shown that the antimicrobial activity of XOS is limited to the gastrointestinal digestion tract and that its effects are greatly reduced in later stages (98). Corncob XOS exhibited an increased digestion and lowering of lipid vacuolization in fish, *Dicentrarchus labrax*, challenged with the pathogen *Aeromonas hydrophila*. Dietary supplementation with XOS also enhanced the serum immunoglobulin level, serum protein content, and lysozyme activity (99). The high intake of these oligosaccharides induces the production of the antimicrobial peptides (AMPs). AMPs are short, positively charged, defense peptides that kill pathogenic microorganisms directly or indirectly. MOS supplementation induced higher transcriptional levels of antimicrobial peptides in the head-kidney and spleen of *Ctenopharyngodon idella* (100, 101).

## Conclusion and Future Prospects

Hemicellulose-derived oligosaccharides offer themselves as a viable and cost-effective alternative to the conventional prebiotics, such as FOS, GOS, and trans-galactooligosaccharides (TOS). Information on the modulation of microbiota and disease recovery of the host upon the ingestion of HDOs is rapidly emerging. The dynamics of microbial diversity and gut environment upon the consumption of HDOs, under both normal and diseased conditions, is being explored at a faster pace. Noteworthy mechanisms delineating the utilization of HDOs, leading to the favorable modulation of gut microbiota, are being unraveled. Knowledge of their interaction with commensal and pathogenic bacteria will lead to the development of a more efficient HDO-based prebiotic formulation that would target the specific gut microbiota for better health.

In spite of a large number of biochemical investigations, well-designed clinical trials are necessary for establishing the specific alteration in gut microbiota at the taxa level by the HDOs. Clinical trials are usually limited to animal models, but can be utilized for deciphering the particular mechanism of action of HDOs in health-promoting activities. *In vitro* studies with particular groups of microorganisms will also be helpful in revealing information about the gut environment. Statistical approaches and machine learning with multi-omics will provide further details and unravel the interactions between the host, microbes, and HDOs.

## Author Contributions

UJ provided the idea and was involved in conceptualization, visualization, writing the original draft, and writing the review and editing. NK and BP were involved in supervision, resources, and writing the review and editing. All authors contributed to the article and approved the submitted version.

## Conflict of Interest

The authors declare that the research was conducted in the absence of any commercial or financial relationships that could be construed as a potential conflict of interest.

## Publisher's Note

All claims expressed in this article are solely those of the authors and do not necessarily represent those of their affiliated organizations, or those of the publisher, the editors and the reviewers. Any product that may be evaluated in this article, or claim that may be made by its manufacturer, is not guaranteed or endorsed by the publisher.
